# A Possible Mode of Action of Methyl Jasmonate to Induce the Secondary Abscission Zone in Stems of *Bryophyllum calycinum*: Relevance to Plant Hormone Dynamics

**DOI:** 10.3390/plants11030360

**Published:** 2022-01-28

**Authors:** Michał Dziurka, Justyna Góraj-Koniarska, Agnieszka Marasek-Ciolakowska, Urszula Kowalska, Marian Saniewski, Junichi Ueda, Kensuke Miyamoto

**Affiliations:** 1 The Franciszek Górski Institute of Plant Physiology, Polish Academy of Sciences, Niezapominajek 21, 30-239 Krakow, Poland; 2 The National Institute of Horticultural Research, Konstytucji 3 Maja 1/3, 96-100 Skierniewice, Poland; justyna.goraj@inhort.pl (J.G.-K.); agnieszkamarasek@wp.pl (A.M.-C.); urszula.kowalska@inhort.pl (U.K.); marian.saniewski@inhort.pl (M.S.); 3 Department of Biological Science, Graduate School of Science, Osaka Prefecture University, 1-1 Gakuen-cho, Naka-ku, Sakai, Osaka 599-8531, Japan; ueda@b.s.osakafu-u.ac.jp; 4 Faculty of Liberal Arts and Sciences, Osaka Prefecture University, 1-1 Gakuen-cho, Naka-ku, Sakai, Osaka 599-8531, Japan

**Keywords:** auxin-related compound, *Bryophyllum calycinum*, indole-3-acetic acid, methyl jasmonate, plant hormone dynamics, secondary abscission

## Abstract

Plants can react to environmental stresses through the abscission of infected, damaged, or senescent organs. A possible mode of action of methyl jasmonate (JA-Me) to induce the formation of the secondary abscission zone (SAZ) in the stems of *Bryophyllum calycinum* was investigated concerning plant hormone dynamics. Internode segments were prepared mainly from the second or third internode from the top of plants with active elongation. JA-Me applied to the middle of internode segments induced the SAZ formation above and below the treatment after 5–7 days. At 6 to 7 days after JA-Me treatment, the above and below internode pieces adjacent to the SAZ were excised and subjected to comprehensive analyses of plant hormones. The endogenous levels of auxin-related compounds between both sides adjacent to the SAZ were quite different. No differences were observed in the level of jasmonic acid (JA), but the contents of 12-oxo-phytodienoic acid (OPDA), a precursor of JA, and *N*-jasmonyl-leucine (JA-Leu) substantially decreased on the JA-Me side. Almost no effects of JA-Me on the dynamics of other plant hormones (cytokinins, abscisic acid, and gibberellins) were observed. Similar JA-Me effects on plant hormones and morphology were observed in the last internode of the decapitated growing plants. These suggest that the application of JA-Me induces the SAZ in the internode of *B. calycinum* by affecting endogenous levels of auxin- and jasmonate-related compounds.

## 1. Introduction

Plants encounter plentiful biotic and abiotic stresses, leading to shedding (separation) of no longer needed or damaged organs such as leaves, branches, flowers, and fruits, from the parent plants. This process is known as abscission, and it is strongly associated with plant growth and development [1,2,3,4,5,6,7,8,9]. In the process of abscission, mechanical weakening of cell walls at the abscission zone is brought about by the degradation of the middle lamella by multiple cell-wall-degrading enzymes such as cellulase, polygalacturonases, pectin methyl esterases, and so forth, resulting in shedding [4,9,10,11,12,13,14,15].

The position and the time of the formation of abscission zones are determined genetically in each organ, and abscission zones once formed commonly do not differentiate further. Contrarily, in response to tissue injury or infection, differentiation of abscission zones in abnormal positions on stems, petioles, pedicles, and branches, designated as the secondary abscission zone (SAZ), can occur in vivo [1,16]. The secondary abscission has been observed primarily in various in vitro systems involving pedicels of *Malus sylvestris* [17] and *Pyrus communis* [18], stems of *Impatiens sultani* [10,11,19], *Morus alba* [20], *Citrus sinensis* [21], and *Phaseolus vulgaris* [16], and petiole explants of *P. vulgaris* [22], *Pisum sativum* pedicle, or *Euphorbia pulcherrima* flower [23].

The SAZs are induced by some signals, especially plant hormone cues, between neighboring cells [8,24,25,26]. According to histological analyses, the formation of the SAZ in the stems of *Bryophyllum calycinum* was characterized by the presence of newly synthesized cell plates resulting from periclinal cell division within one layer of mother cells in stems [27].

Plant hormones are well known to play an essential role in plant growth and development, including the abscission or induction of transdifferentiation in mature cortical cells. Ueda et al. [28,29] reported that jasmonic acid (JA) and methyl jasmonate (JA-Me) as senescence-promoting substances promoted the abscission of bean petiole explants. JA-Me also promotes leaf abscission in intact *Kalanchoe blossfeldiana* [30] and *B. calycinum* plants [31]. Furthermore, Saniewski et al. [31] have reported that JA-Me at a concentration of 0.5% (*w*/*w*) applied as a lanolin paste in different stem explants or the debladed petiole induced the formation of the SAZs in *B. calycinum*. These suggest that JA and JA-Me (designated as jasmonates, JAs) have a powerful effect of inducing the SAZ and developing an abscission zone that has already been initiated in plant tissues, resulting in leaf abscission. Ito and Nakano [32] have suggested that a decrease in auxin levels might be considered to provide the first signal for abscission in pedicel abscission in tomatoes. In the stem of *B. calycinum,* indole-3-acetic acid (IAA) applied to a decapitated shoot or internode explants totally prevented the formation of the SAZ in the stems induced by JA-Me [31,33]. However, it should be mentioned that only IAA application substantially induces the formation of the SAZ not only in internode explants, petiole segments, and the petiole after excision of the leaf blade but also in decapitated stems in intact plants of *B. calycinum* [31,33]. It is suggested that in mechanisms of the SAZ formation induced by exogenously applied IAA in the internode of *B. calycinum*, an auxin gradient is vital, and the gradient results from polar IAA transport from the application site [27,31,33]. However, those phenomena induced by JA-Me have not been reported.

As mentioned earlier, plants belonging to the Crassulaceae family show fascinating phenomena, such as leaf abscission and secondary abscission zone formation, easily inducted. This was the reason we chose for experiments the important medicinal plant *Bryophyllum calycinum* (syn. *Kalanchoe pinnata*) [34].

To clarify JA-Me’s possible mode of action to induce the formation of the SAZ in terms of its plant hormone dynamics, we focused on differences in plant hormone dynamics between adjacent tissues to the SAZ induced by JA-Me in stem segments, as well as decapitated growing plants of *B. calycinum.* In this paper, comprehensive analyses of plant hormones in JA-Me treated stems, mainly internodes segments, of *B. calycinum* were reported.

## 2. Results

### 2.1. The Effect of JA-Me on Induction of the Secondary Abscission in Internode Segments and Decapitated Plants of Bryophyllum calycinum

**Figure 1:**
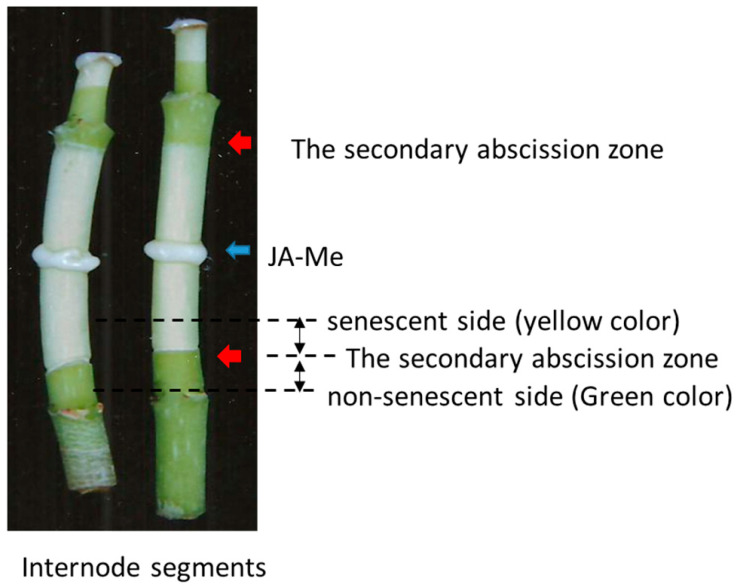
Secondary abscission zone (SAZ) induced by the application of methyl jasmonate (JA-Me) in internode segments of *Bryophyllum calycinum*. The treatment was made in the middle of internode explants. Photograph was taken 8 days after treatment. Red and blue arrows indicate the SAZ and JA-Me treatment place, respectively. Stem pieces (ca. 3–4 mm in length) above and below the SAZ were subjected to comprehensive plant hormone analyses.

In *Bryophyllum calycinum*, JA-Me application (0.5%; *w*/*w* in lanolin) to the middle of internode segments induced formation of the SAZ, observed at length from 0.5 to 2 cm above and below the JA-Me treatment, 5–7 days after the treatment (Figure 1). JA-Me application induced senescence or loss of chlorophylls in the internode segments in both acropetal and basipetal directions. Treatment with JA-Me (0.5%, *w*/*w* in lanolin) at the middle of the last internode in decapitated growing plants also induced the SAZ below the treatment (Supplementary Figure S1).

### 2.2. Changes in the Levels of Endogenous Plant Hormones in Relation to the Formation of the Secondary Abscission Zone Induced by JA-Me

Comprehensive analyses of the endogenous plant hormones and their related compounds concerning the induction of the SAZ were performed in the internode segments of *B. calycinum*. At the appropriate time or 6 or 7 days after the treatment, small pieces of the internode segments adjacent to the SAZ were harvested for plant hormone analyses, as illustrated in Figure 1. Similar internode pieces above and below the SAZ in decapitated growing plants of *B. calycinum* were also subjected to the plant hormone analyses (Supplementary Figure S1).

#### 2.2.1. Effect of JA-Me on Auxin-Related Compounds

**Figure 2:**
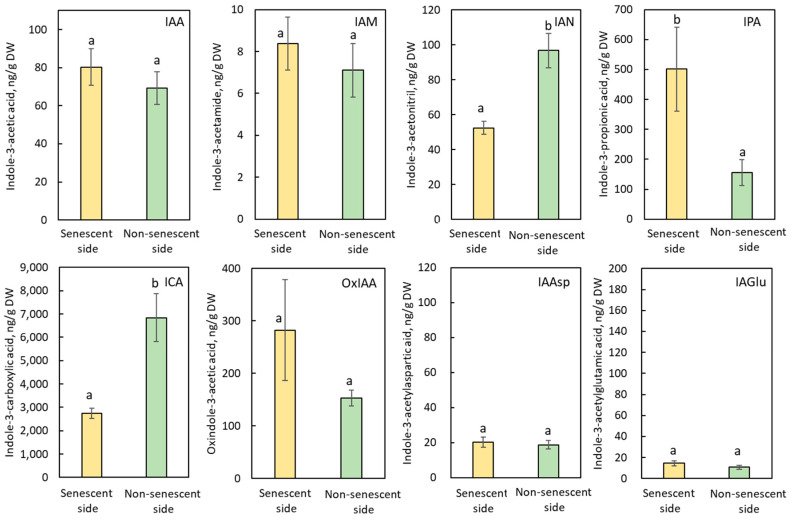
Endogenous levels of auxin-related compounds in the senescent and non-senescent sides of the SAZ induced by JA-Me in the internode explants of *Bryophylum calycinum*. IAA: indole-3-acetic acid; IAM: in-dole-3-acetamide; IAN: indole-3-acetonitrile; IPA: indole-3-propionic acid; ICA: indole-3-carboxylic acid; OxIAA: 2-oxindole-3-acetic acid; IAAsp: indole-3-acetylaspartic acid; IAGlu: indole-3-acetylglutamic acid. Values are the mean with standard error (n = 6). Different letters on the column (a, b) indicated statistically significant at *p* < 0.05 after ANOVA.

As shown in Figure 2, the following auxins and their related compounds were successfully identified in internode segments of *B. calycinum*: indole-3-acetic acid (IAA), indole-3-acetamide (IAM), indole-3-acetonitrile (IAN), 2-oxindole-3-acetic acid (OxIAA), indole-3-carboxylic acid (ICA), indole-3-acetyl-aspartic acid (IAAsp), indole-3-acetyl-glutamic acid (IAGlu), and indole-3-propionic acid (IPA).

In the internode segment treated with JA-Me, endogenous levels of IAA, IAGlu, IAAsp, OxIAA, and IAM, in the above (senescent side, yellow color) and below pieces (non-senescent side, green color) adjacent to the secondary abscission in internode segments were similar. However, the contents of IAN and ICA were lower in the senescent than in the non-senescent side (Figure 2). These results suggest that the SAZ formation induced by JA-Me is closely related to the modification of IAA biosynthetic pathways via IAM, IAN, and ICA from tryptophan.

It should be mentioned that the endogenous level of IPA is much higher in the senescent than in the non-senescent side, suggesting that IAA metabolism to IPA is possible to be related to the SAZ induced by JA-Me (Figure 2).

Similar results of the effect of JA-Me on the endogenous levels of auxin-related compounds in the internode segments were obtained in the last internode of decapitated growing plants of *B. calycinum* (Supplementary Figure S2). These results suggest that the application of JA-Me substantially affects the IAA metabolism in the internode of *B. calycinum* and then might induce secondary abscission.

#### 2.2.2. Effect of JA-Me on Jasmonate-Relating Compounds, Abscisic Acid, Salicylic Acid and Benzoic Acid

**Figure 3:**
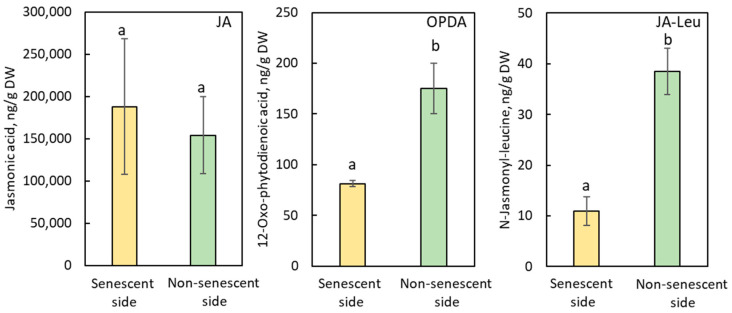
Endogenous levels of jasmonate-related compounds in the senescent and non-senescent sides of the SAZ induced by JA-Me in the internode explants of *Bryophyllum calycinum*. JA: jasmonic acid; OPDA: 12-oxo-phytodienoic acid; JA-Leu: *N*-jasmonyl-leucine. Values are the mean with standard error (n = 6). Different letters on the column (a, b) indicated statistically significant at *p* < 0.05 after ANOVA.

The contents of 12-oxo-phytodienoic acid (OPDA) and *N*-jasmonyl-leucine (JA-Leu) were substantially lower in the stem above the senescent than in the non-senescent side, but the content of jasmonic acid (JA) was similar in the stem pieces below and above the SAZ (senescent and non-senescent) in the internode explants (Figure 3).

An almost similar tendency was observed in the decapitated growing plants of *B. calycinum* (Supplementary Figure S3), suggesting that the application of JA-Me substantially increases endogenous levels of JA.

**Figure 4:**
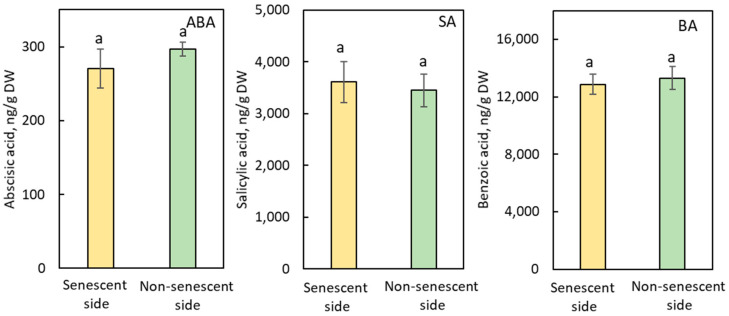
Endogenous levels of abscisic acid (ABA), salicylic acid (SA), and benzoic acid (BA) in the senescent and non-senescent sides of the SAZ induced by JA-Me in the internode explants of *Bryophylum calycinum*. Values are the mean with standard error (n = 6). Different letters on the column (a) indicated statistically significant at *p* < 0.05 after ANOVA.

The endogenous levels of abscisic acid (ABA), salicylic acid (SA), and benzoic acid (BA) occurred in similar amounts in the stem pieces below and above the SAZs induced by JA-Me both in stem explants and in the internode of decapitated growing plants of *B. calycinum* (Figure 4 and Supplementary Figure S4).

#### 2.2.3. Effect of JA-Me on Cytokinins

**Figure 5:**
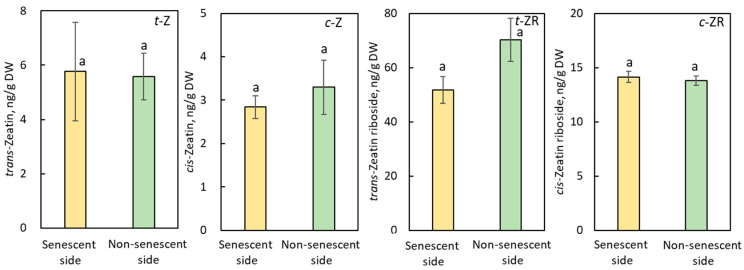
Endogenous levels of cytokinins in the senescent and non-senescent sides of the SAZ induced by JA-Me in the internode explants of *Bryophylum calycinum*. t-Z: *trans*-Zeatin; c-Z: *cis*-Zeatin; t-ZR: *trans*-Zeatin riboside; c-ZR: *cis*-Zeatin riboside. Values are the mean with standard error (n = 6). Different letters on the column (a) indicated statistically significant at *p* < 0.05 after ANOVA.

The contents of identified cytokinins such *trans*-zeatin (t-Z), *cis*-zeatin (c-Z), *trans*-zeatin riboside (t-ZR), and *cis*-zeatin riboside (c-ZR) were similar in the senescent and non-senescent sides of SAZ induced by JA-Me both in internode explants and in the internode of decapitated growing plants of *B. calycinum* (Figure 5 and Supplementary Figure S5).

#### 2.2.4. Effect of JA-Me on Gibberellins

Thirteen gibberellins (GAs), gibberellin A_1_ (GA_1_), GA_3_, GA_4_, GA_5_, GA_6_, GA_7_, GA_8_, GA_9_, GA_15_, GA_19_, GA_20_, GA_44_, and GA_53_, were also successfully identified in the internode segments of *B. calycinum*. Similar levels of these GAs were found in both the senescent and non-senescent sides of SAZ induced by JA-Me, except that GA_8_ was lower above the SAZ (senescent side; Table 1). A similar tendency was observed in the decapitated growing plants of *B. calycinum* treated with JA-Me (Supplementary Table S1).

## 3. Discussion

As mentioned in the Introduction (Section 1), many plant species develop the secondary abscission zone that extends between organs and the main body of the plants to shed. Plant hormones may play an essential role in the transdifferentiation in mature cortical cells to induce the SAZ. JA-Me and JA (designated as jasmonates, JAs) show the powerful effect of inducing the SAZ in stems and developing an abscission zone that has already been initiated in plant tissues in *B. calycinum* [31,33]. JAs were applied in lanolin paste, where lanolin alone did not affect morphological changes in the internode segments. This situation was demonstrated in previous works [27,31,35]. In the stem of *B. calycinum*, IAA applied to a decapitated shoot or internode explants prevented the formation of the SAZ induced by JA-Me [31,33]. Contrarily, IAA application has also been demonstrated to substantially induce the formation of the SAZ not only in internode explants, petiole segments, and petiole after excision of the leaf blade in intact plants but also decapitated stems in intact plants of *B. calycinum* [31,33]. A decrease in auxin levels might be considered as providing the first signal for abscission, as suggested in Arabidopsis [36] and tomatoes [32,37]. JAs, together with the disruption of endogenous auxin status by the decapitation or excision, may trigger the formation of the SAZ. The results confirm our previous observations [27,31,33], indicating that JA-Me is translocated in stem explants of *B. calycinum* in both ways, acropetally and basipetally, from the place of treatment. The SAZ development place is considered the final result of the stem’s secondary abscission formation and senescence. Thus, it could be asserted that fresh, green tissues of the stem below the SAZ are not affected by JA-Me and can also be treated as a control.

What kinds of hormonal control factors are responsible for the formation of the SAZ induced by JA-Me? The SAZ formation by JA-Me has already been reported to be closely related to auxins [27,31]. Therefore, it is worthwhile to study the dynamics of plant hormones, especially auxins in the senescent and the non-senescent sides of the SAZ induced by JA-Me. Notably, the IAA gradient was not observed in the explants between the induced SAZ on both sides. The same situation occurred in the internode of the decapitated growing plant (Figure 2 and Supplementary Figure S2).

It has been reported that JA-Me is converted into JA and jasmonyl-isoleucine (JA-Ile), activating the jasmonates signaling pathway and emission of volatile organic compounds in *Achyranthes bidentate* [38]. The application of JA-Me resulted in the differences in endogenous levels of auxin-related compounds such IAN, ICA, and IPA in the senescent and non-senescent sides of the SAZ (Figure 2 and Supplementary Figure S2). Endogenous levels of OxIAA, which is one of IAA metabolites, were also different. These results suggest that the SAZ induced by JA-Me is closely related to the disruption of IAA metabolism in the stem adjacent to the SAZ.

IPA and IBA, other auxins that share similar structural scaffolds, are strongly conjugated and hydrolyzed with enzymes with similar or even higher activities than with IAA or IAA conjugates [39]. The occurrence of IBA has been reported in various plants, including *B. calycinum* [27]. In the present study, we report for the first time the occurrence of IPA in *B. calycinum*. The natural occurrence of IPA is scant, and until now, little is known about the physiological activity of IPA compared to IAA [39]. The content of IPA was relatively high in the stem of *B. calycinum*, and evidently, the content of IPA further increased on the stem side of JA-Me treatment, suggesting that IPA is responsible for the SAZ formation in *B. calycinum*.

Jasmonates (JAs) might function as a core signal in the plant hormone signaling network, a signal of JAs interacting with other hormone signaling to regulate plant growth, and abiotic and biotic stress tolerance [40,41,42,43]. Evidence for a close functional relationship between JAs signaling and auxin homeostasis has been well documented [44,45]. Du et al. [46] showed that biosynthesis and signaling of JA and IAA are differentially regulated by different abiotic stresses in rice, suggesting that the balance between JA and IAA homeostasis and their signaling are critical for plant development and stress responses. The application of JA-Me substantially induces an increase in the endogenous levels of JA in the stem of explant and internode of the decapitated growing plant of *B. calycinum*, as well as the disruption of auxin metabolism, but negligibly affected dynamics of ABA, cytokinins, and GAs (Figure 3, Figure 4 and Figure 5, Table 1, Supplementary Figures S3–S5 and Table S1). Thus, cross-talk between JAs and auxin might be essential for the induction of the SAZ formation.

Based on the results of comprehensive analyses of endogenous plant hormones, Marasek-Ciolakowska et al. [27] strongly suggested that GAs and cytokinins did not contribute to the formation of the IAA-induced SAZ in *B. calycinum*. In this experiment, JA-Me also little affected the endogenous level of GAs, ABA, and cytokinins in stems above and below the SAZ (Figure 3 and Figure 4, Table 1, Supplementary Figures S3 and S4, and Table S1). Thus, these plant hormones seem not to contribute to the formation of the JA-Me-induced SAZ in *B. calycinum* as the IAA-induced one [27].

Until now, four tryptophan (Trp)-dependent pathways of IAA biosynthesis, namely the indole-3-acetamide (IAM) pathway, the indole-3-pyruvic acid (IPyr) pathway, the tryptamine pathway, and the indole-3-acetaldoxime (IAOx) pathway, were identified in plants [39,47,48,49,50], although biosynthesis pathway(s) of IAA in plants of the Crassulaceae family (succulents) is unknown. The Trp-independent IAA biosynthesis from indole was also documented in some plants [50]. In *Arabidopsis thaliana*, indole-3-carbaldehyde and indole-3-carboxylic acid (ICA) are synthesized from Trp via intermediates such IAOx and IAN, although ICA can also be attributed to the degradation of IAA [51]. Whether ICA can be converted to IPA and vice versa, as indole-3-butyric acid (IBA) and IAA interconversions, has not been shown as yet [39]. ICA has been identified in *Pinus sylvestris* needles, in the leaves of *Ginkgo biloba*, and in the stem of *B. calycinum* [27,31,52].

The occurrence of IAM and IAN in the stem of *B. calycinum* may suggest that biosynthesis of IAA in the plant is going through the IAM and IAOx pathways since IAM and IAN are downstream intermediate metabolites of IAOx [50,53]. The IAOx-dependent IAA biosynthesis pathway was indicated in some plants, but it is not a common pathway [50]. Other pathways of IAA biosynthesis are also possible in *B. calycinum*. Intensive studies on JAs-dependent changes in metabolism or biosynthesis of IAA and the physiological function of ICA, related to the secondary abscission formation, will be needed in the future.

## 4. Materials and Methods

### 4.1. Plant Materials and Hormone Treatment

Three- to six-month-old plants of *Bryophyllum calycinum* Salisb. (Crassulaceae), propagated from epiphyllous buds arising in the marginal notches of the leaves, were used in the experiments. Stem segments and decapitated stems of growing *B. calycinum* plants were used in methyl jasmonate (JA-Me) treatment.

Internode segments at the length of ca. 4–5 cm with two nodes (leaves removed) were excised, from mainly the second or third internodes from the top of growing plants with active elongation. The segments were treated with JA-Me at 0.5% (*w*/*w*) in lanolin paste in the middle of the internode and kept vertically in a 50 mL glass chamber with moistened papers at the bottom of these explants under natural light conditions in a greenhouse, as shown in Figure 1. In June, August, and September, experiments were repeated three times with 15 to 20 explants.

A similar experiment with decapitated growing plants was carried out. After decapitation of the apical part of the growing plant shoot, JA-Me (0.5%, *w*/*w* in lanolin) was applied in the middle of the last internode, as shown in Supplementary Figure S1. The experiment was repeated twice from August to October with 20 explants.

### 4.2. Analyses of Plant Hormones in Relation to the Formation of the Secondary Abscission Zone Induced by Methyl jasmonate

Analyses of plant hormones were performed according to the methods reported previously [27,35,54,55,56]. At 6 to 7 days after treatment with JA-Me, the below (non-senescent, green) and above (senescent, yellow) parts of ca. 3–4 mm internode pieces adjacent to the SAZ formed by JA-Me application in the stem of *B. calycinum* were excised, respectively. Excised samples were immediately frozen in liquid N_2_ and then lyophilized. Lyophilized materials in each piece of internode were combined, and an aliquot of a small amount (ca. 10 mg DW) was used for comprehensive plant hormone analyses. Lyophilized materials with appropriate amounts of a mixture of each stable isotope-labeled plant hormone as an internal standard were extracted with an organic solvent consisting of methanol/water/formic acid = 15: 4: 1 (*v*/*v*/*v*) three times. Respective extracts were combined and then evaporated under N_2_. The extract obtained was re-suspended in 3% methanol in 1 M formic acid and then cleaned up on hybrid SPE cartridges (BondElut Plexa PCX, Agilent, Santa Clara, CA, USA). Qualitative and quantitative analyses of plant hormones and other related compounds were performed on a HPLC-MS/MS system with UHPLC apparatus (Agilent Infinity 1260, Agilent, Waldbronn, Germany) coupled to a triple quadruple mass spectrometer ESI-MS/MS (6410 Triple Quad LC/MS, Agilent, Santa Clara, CA., USA). Plant hormones were separated on an Ascentis Express RP-Amide analytical column (particle size: 2.7 μm; 2.1 mm × 150 mm; Supelco, Bellefonte, PA., USA) at 60 °C, at a linear gradient of water vs. acetonitrile both with 0.01% of formic acid. As internal standards, [^15^N_4_] dihydrozeatin, [^15^N_4_] kinetin, [^2^H_5_] *trans*-zeatin riboside (t-ZR), [^2^H_5_] indole-3-acetic acid (IAA), [^2^H_4_] indole-3-acetonitrile, [^2^H_4_] salicylic acid (SA), [^2^H_2_] gibberellin A_1_ (GA_1_), [^2^H_2_] gibberellin A_4_ (GA_4_), [^2^H_2_] gibberellin A_5_ (GA_5_), [^2^H_2_] gibberellin A_6_ (GA_6_), [^2^H_6_] *cis*, *trans*-abscisic acid (ABA), [^2^H_5_] benzoic acid (BA), [^2^H_5_] jasmonic acid (JA), and [^2^H_5_] dinor-12-oxo-phytodienoic acid (dinor-OPDA) were used. All standards, except for [^2^H_5_] JA supplied by CND Isotopes (Quebeck, Canada) and [^2^H_5_] dinor OPDA supplied by Cayman Chem. Comp. (Ann Arbor, USA), were from OlChemim (Olomouc, Czech Republic) at the highest available purity. Multiple reaction monitoring (MRM) transitions were used to identify and quantify all compounds of interest. Quantitation was based on calibration curves obtained with each pure standard compound taking account of the recovery rates of an internal standard used. Further technical details are given by the references cited above.

### 4.3. Statistical Analysis

The analysis of variance (ANOVA) was conducted using STATISTICA software (StatSoft, Kraków, Poland). To compare the means, Duncan’s multiple range test was used. Values of *p* < 0.05 were considered to be statistically significant. Values are expressed as the mean with standard error. Different letters in the columns in the figures and tables indicate statistical differences.

## 5. Conclusions

A comprehensive study of the dynamics of plant hormones in the stem pieces above and below the SAZ induced by the application of JA-Me in *B. calycinum* revealed that the application of JA-Me substantially affected auxin metabolism and the endogenous status of JAs. However, it negligibly affected the endogenous IAA levels. These suggest that the mode of JA-Me action to induce the SAZ is different from that of IAA, whereas IAA also induces the SAZ. JA-Me functions as a trigger modifying metabolism of IAA and JAs to induce the formation of the SAZ in the stem of *B. calycinum*.

## Figures and Tables

**Table 1:** Endogenous levels of gibberellins in the senescent and non-senescent sides of the SAZ, induced by JA-Me in the internode explants of *Bryophylum calycinum*. Values are the mean with standard error (n = 6). Different letters (a, b) on the column indicated statistically significant at *p* < 0.05 after ANOVA.

	Endogenous Levels (ng/g DW)	
	Senescent Side	Non-Senescent Side
Gibberellin A_1_	36.26 ± 1.52 a	35.88 ± 1.11 a
Gibberellin A_3_	4785.54 ± 382.88 a	5420.84 ± 420.90 a
Gibberellin A_4_	54.37 ± 25.21 a	35.49 ± 20.50 a
Gibberellin A_5_	61.05 ± 7.16 a	57.94 ± 19.21 a
Gibberellin A_6_	547.32 ± 25.36 a	595.66 ± 44.81 a
Gibberellin A_7_	54.37 ± 9.60 a	35.49 ± 20.50 a
Gibberellin A_8_	23.71 ± 9.47 a	74.10 ± 6.85 b
Gibberellin A_9_	65.62 ± 4.20 a	62.66 ± 3.40 a
Gibberellin A_15_	1.23 ± 0.31 a	2.02 ± 0.42 a
Gibberellin A_19_	61.30 ± 2.87 a	63.42 ± 4.55 a
Gibberellin A_20_	83.55 ± 11.62 a	125.63 ± 32.06 a
Gibberellin A_44_	61.49 ± 2.17 a	59.94 ± 1.97 a
Gibberellin A_53_	98.90 ± 16.75 a	87.910 ± 12.97 a

## Data Availability

The data sets generated for this study are available in this article and Supplementary Material.

## Supplementary Materials

The following are available online at https://www.mdpi.com/article/10.3390/plants11030360/s1, Figure S1: The secondary abscission zone induced by the application of methyl jasmonate (JA-Me) in the last internode of decapitated growing plants of *Bryophyllum calycinum*, Figure S2: Endogenous levels of auxin-related compounds in the stem pieces above and below the secondary abscission zone induced by JA-Me in the last internode of decapitated growing plants of *Bryophyllum calycinum*, Figure S3: Endogenous levels of jasmonate-related compounds in the stem pieces above and below the secondary abscission zone induced by JA-Me in the last internode of decapitated growing plants of *Bryophyllum calycinum*. Figure S4: Endogenous levels of abscisic acid, salicylic acid, and benzoic acid in the stem pieces above and below the secondary abscission zone induced by JA-Me in the last internode of decapitated growing plants of *Bryophyllum calycinum*. Figure S5: Endogenous levels of cytokinins in the stem pieces above and below the secondary abscission zone induced by JA-Me in the last internode of decapitated growing plants of *Bryophyllum calycinum*. Table S1: Endogenous levels of gibberellins in the stem pieces above and below the secondary abscission zone induced by JA-Me in the last internode of decapitated growing plants of *Bryophyllum calycinum*.
