# Supplementary material for: A Possible Mode of Action of Methyl Jasmonate to Induce the Secondary Abscission Zone in Stems of Bryophyllum calycinum: Relevance to Plant Hormone Dynamics
Source: Plants (Basel). 2022 Jan 28;11(3):360. doi: 10.3390/plants11030360 (PMC8840011; doi:10.3390/plants11030360)
Supplement: Supplementary file 1 [file plants-11-00360-s001.zip › plants-1531548-supplementary.pdf]

plants

Supplementary material for:

A Possible Mode of Action of Methyl Jasmonate to Induce the Secondary Abscission Zone in Stems of *Bryophylum calycinum*: Relevance to Plant Hormone Dynamics

Michał Dziurka [1,*], Justyna Góraj-Koniarska [2], Agnieszka Marasek-Ciolakowska [2], Urszula Kowalska [2], Marian Saniewski [2], Junichi Ueda [3], Kensuke Miyamoto [4,*]

Citation: Dziurka M., Góraj-Koniarska J., Marasek-Ciolakowska A., Kowalska U., Saniewski M., Ueda J., Miyamoto K. A Possible Mode of Action of Methyl Jasmonate to Induce the Secondary Abscission Zone in Stems of Bryophyllum calyci-num: Relevance to Plant Hormone Dynamics . *Plants* **2022**, *11*, 360. https://doi.org/10.3390/plants11030360

Academic Editor: Tae-Hwan Kim

Received: 14 December 2021
Accepted: 25 January 2022
Published: 28 January 2022

Publisher's Note: MDPI stays neutral with regard to jurisdictional claims in published maps and institutional affiliations.

Copyright: © 2022 by the author. Licensee MDPI, Basel, Switzerland. This article is an open access article distributed under the terms and conditions of the Creative Commons Attribution (CC BY) license (https://creativecommons.org/licenses/by/4.0/).

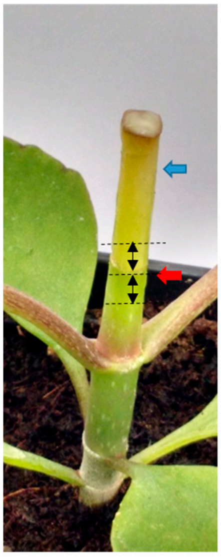

Figure S1. The secondary abscission zone induced by the application of methyl jasmonate (JA-Me) in the last internode of decapitated growing plants of *Bryophylum calycinum*. The treatment was made in the last internode of decapitated plants. Photograph was taken 8 days after treatment. Red and blue arrows indicate the place of the secondary abscission zone and JA-Me treatment, respectively. Stem pieces (ca. 3-4 mm in length) above and below the secondary abscission zone were subjected to the comprehensive plant hormone analyses.

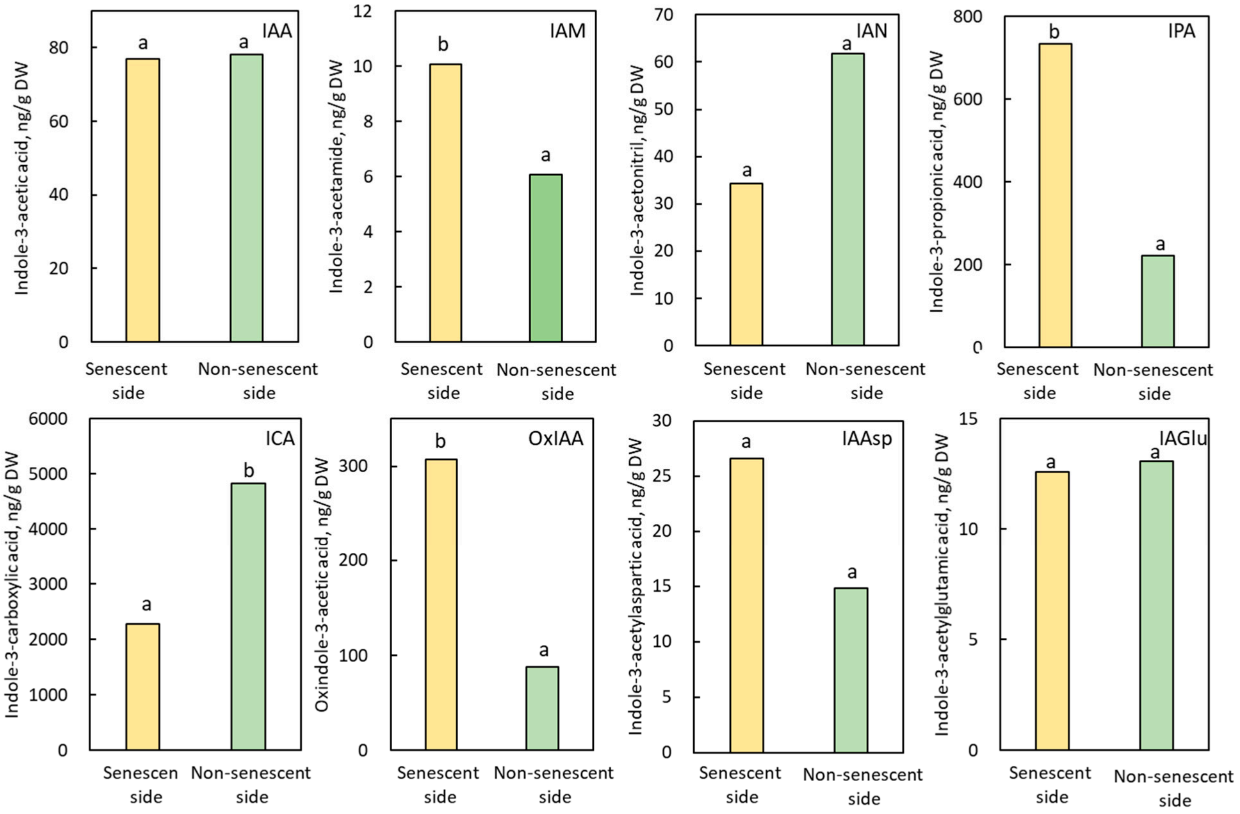

Figure S2. Endogenous levels of auxin-related compounds in the senescent and non-senescent sides of the secondary abscission zone (SAZ) induced by JA-Me in the last internode of decapitated growing plants of *Bryophylum calycinum*. IAA: indole-3-acetic acid; IAM: indole-3-acetamide; IAN: indole-3-acetonitrile; IPA: indole-3-propionic acid; ICA: indole-3-carboxylic acid; OxIAA: 2-oxindole-3-acetic acid; IAAsp: indole-3-acetylaspartic acid; IAGlu: indole-3-acetylglutamic acid. Values are the mean with n=2. Different letters on the column (a, b) indicated statistically significant at $P < 0.05$ after ANOVA.

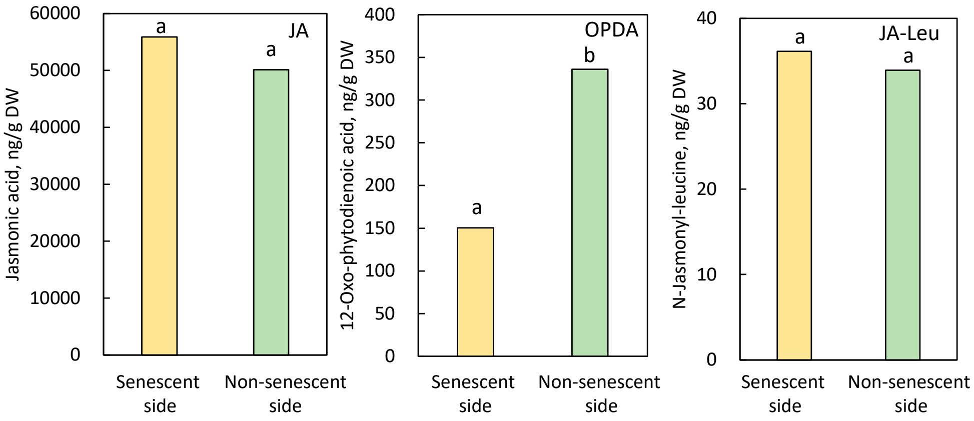

Figure S3. Endogenous levels of jasmonate-related compounds in the senescent and non-senescent sides of the SAZ induced by JA-Me in the last internode of decapitated growing plants of *Bryophylum calycinum*. JA: jasmonic acid; OPDA: 12-oxo-phytodienoic acid; JA-Leu: N-jasmonyl-leucine. Values are the mean with n=2. Different letters on the column (a, b) indicated statistically significant at P < 0.05 after ANOVA.

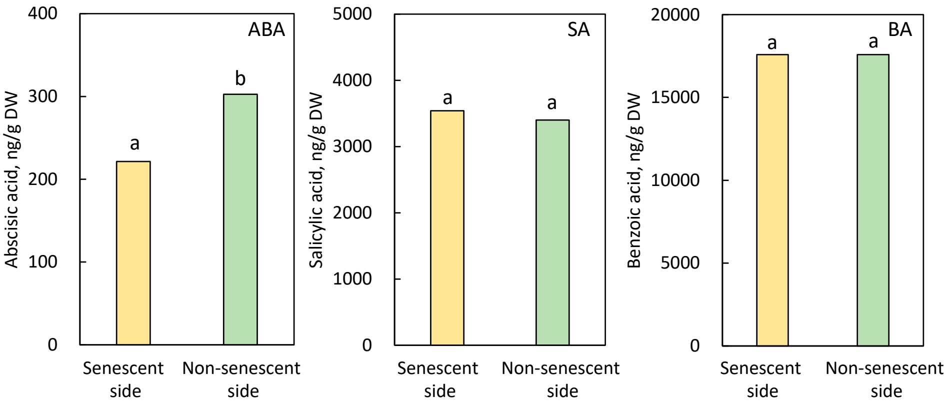

Figure S4. Endogenous levels of abscisic acid (ABA), salicylic acid (SA), and benzoic acid (BA) in the senescent and non-senescent sides of the SAZ induced by JA-Me in the last internode of decapitated growing plants of *Bryophylum calycinum*. Values are the mean with n=2. Different letters on the column (a, b) indicated statistically significant at P < 0.05 after ANOVA.

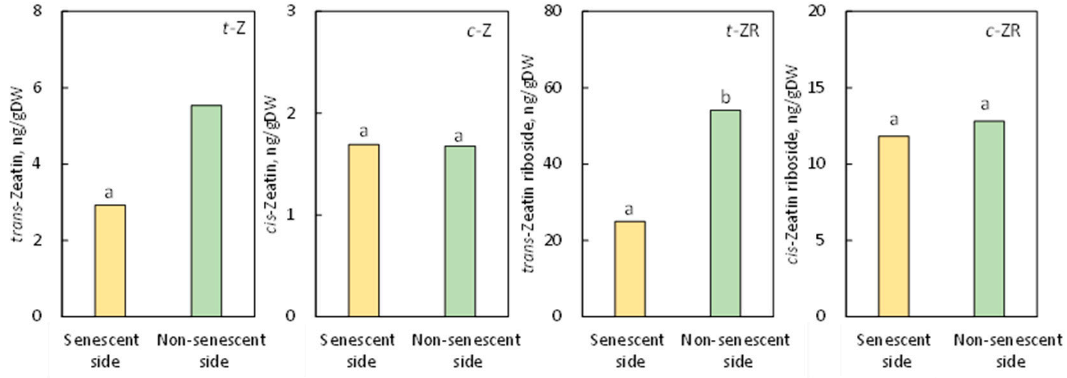

Figure S5. Endogenous levels of cytokinins in the senescent and non-senescent sides of the SAZ induced by JA-Me in the last internode of decapitated growing plants of *Bryophylum calycinum*. *t*-Z: *trans*-Zeatin; *c*-Z: *cis*-Zeatin; *t*-ZR: *trans*-Zeatin riboside; *c*-ZR: *cis*-Zeatin riboside. Values are the mean with n=2. Different letters on the column (a, b) indicated statistically significant at P < 0.05 after ANOVA.

Table S1. Endogenous levels of gibberellins in the senescent and non-senescent sides of the SAZinduced by JA-Me in the last internode of decapitated growing plants of *Bryophylum calycinum*. Values are the mean with n=2. Different letters (a, b) on the column indicated statistically significant at P<0.05 after ANOVA.

	**Endogenous levels (ng/g DW)**	
	**Senescent side**	**Non-senescent side**
Gibberellin A_1	34.7 a	34.2 a
Gibberellin A_3	2818.1 a	2594.1 a
Gibberellin A_4	46.0 a	49.4 a
Gibberellin A_5	85.7 a	35.0 a
Gibberellin A_6	783.0 a	707.3 a
Gibberellin A_7	52.7 a	49.9 a
Gibberellin A_8	36.8 a	60.6 a
Gibberellin A_9	75.8 a	57.3 a
Gibberellin A_{15}	1.7 a	2.7 a
Gibberellin A_{19}	67.4 a	57.4 a
Gibberellin A_{20}	106.9 a	147.9 a
Gibberellin A_{44}	46.0 a	49.4 a
Gibberellin A_{53}	130.4 a	47.4 a
